# The impact of thyroid function on intrauterine insemination outcome - a retrospective analysis

**DOI:** 10.1186/1477-7827-12-28

**Published:** 2014-04-05

**Authors:** Birgit Jatzko, Elisabeth Vytiska-Bistorfer, Alexandra Pawlik, Regina Promberger, Klaus Mayerhofer, Johannes Ott

**Affiliations:** 1 Department of Obstetrics and Gynecology, Clinical Department of Gynecologic Endocrinology and Reproductive Medicine, Medical University of Vienna, Waehringer Guertel 18-20, 1090 Vienna, Austria; 2 Department of Surgery, Medical University of Vienna, Waehringer Guertel 18-20, 1090 Vienna, Austria

**Keywords:** Intrauterine insemination, Hypothyroidism, Autoimmune thyroiditis, TSH, Target value

## Abstract

**Background:**

Hashimoto’s thyroiditis is the most common endocrinopathy in premenopausal women, and is associated with various gynecological problems, including recurrent miscarriage and unexplained infertility. A possible influence of Hashimoto’s thyroiditis on the success of intrauterine insemination seems likely, but has not been evaluated as yet. Therefore, the aim of our study was to retrospectively analyze the impact on intrauterine insemination outcome of thyroid function and markers suggestive for Hashimoto’s thyroiditis.

**Methods:**

Retrospective cohort study in a tertiary care center of 540 women who underwent Intrauterine Insemination. The clinical pregnancy rate was the main outcome parameters. The following possible influencing factors were tested: thyroid-stimulating hormone (TSH); thyroid autoantibodies; age; body mass index; type of sterility (primary/secondary); parity; male factor; presence of PCO syndrome; ovulation induction; ovarian stimulation; and current thyroid medication.

**Results:**

The overall clinical pregnancy rate was 6.9% (37/540). Age, thyroid hormone supplementation for thyroid-stimulating hormone (TSH) levels > 2.5 micro-IU/ml, and ovulation induction with HCG were significantly predictive in the multivariate analysis (p < 0.05) as influencing factors for the pregnancy rate after intrauterine insemination.

**Conclusions:**

Women undergoing intrauterine insemination seem to benefit from a strict thyroid hormone supplementation regimen in order to achieve lower TSH levels.

## Background

Intrauterine insemination (IUI) is a simple treatment option for patients with infertility. In detail, a 0.2-0.5 ml processed sperm suspension is deposited with a small catheter transcervically in the uterus, usually without imaging guidance [1]. Clomifene citrate (CC) is often used for ovarian stimulation, and Human Choriogonadotropin (HCG) can be administered intramuscularly to induce ovulation. IUI results in mean pregnancy rates of 10-20% per patient. However, it can vary individually from 5-70%, depending on the indication, which might often be infertility of unknown origin (unexplained infertility) or slightly poor sperm quality [2].

Hashimoto’s thyroiditis (HT), a chronic autoimmune thyroiditis, is the most common endocrinopathy in premenopausal women in developed countries, with an incidence of 5-10%, and is the most frequent cause of subclinical hypothyroidism in women in iodine-sufficient areas. Worldwide, iodine deficiency is still the most prevalent cause of thyroid dysfunction [3,4]. Typically, these women show elevated levels of antibodies against thyroperoxidase (TPO-Ab). Various studies have demonstrated that HT is associated with various gynecological problems. These include recurrent miscarriage [5-12]; otherwise unexplained infertility, defined by the absence of pregnancy after one year of sexual intercourse without contraception [12]; *in vitro* fertilization failure [13]; and resistance against CC stimulation in women with polycystic ovary syndrome (PCO-S) [14].

Moreover, the relationship between thyroid dysfunction and negative outcomes for the mother in terms of miscarriage, preterm delivery and preeclampsia as well as for child in terms of decreased intelligence quotient and birth weight is relatively well known [15,16]. Even subclinical hypothyroidism without thyroid autoantibodies seems to be associated with the above mentioned problems [16-19]. Treatment of overt hypothyroidism during pregnancy is, therefore, mandatory and consists of levothyroxine therapy adjusted to achieve normal trimester-specific serum levels of thyroid stimulating hormone (TSH). In addition to the fact that recommendations for treatment of subclinical hypothyroidism before and during pregnancy differ among professional organisations, a vigorous debate is ongoing on the pros and cons of universal screening for thyroid disease during pregnancy versus targeted case finding [20].

The influence of HT and thyroid hypofunction on IUI success has never been examined. We, thus, aimed to retrospectively evaluate a possible association between TPO-Ab levels and thyroid function parameters with regard to IUI outcome.

## Methods

In a retrospective cohort study, we included all 540 patients who underwent an IUI at the Clinical Department of Gynecologic Endocrinology and Reproductive Medicine of the Medical University of Vienna, Austria, from January 2008 to December 2011. None of the women revealed any additional severe endocrine diseases including adrenogenital syndrome, Cushing’s syndrome and others. Thus, no patients had to be excluded. The study was approved by the Institutional Review Board of the Medical University of Vienna (IRB number: 1751/2012). The indications, the whole proceeding and the assessment for the quality of semen were in accordance with the international guidelines for IUI [1].

### Outcome parameters and evaluated predictive factors

Data were retrieved by retrospective chart review. The primary outcome parameter after IUI was the (clinical) pregnancy rate diagnosed by ultrasound (positive heart rate). The following parameters were analyzed as possible influencing factors on IUI outcome: TSH; TPO-Ab; thyroglobulin antibodies (TG-Ab); patients’ age; body mass index (BMI); type of sterility (primary/secondary); parity; male factor; presence of PCO syndrome; metformin treatment for PCO syndrome; ovulation induction with HCG; CC stimulation; and current thyroid medication due to either (i) overt hypothyroidism (defined as TSH and (total) levothyroxin (T4) > 5.0 μIU/ml and < 64.5 nmol/l, respectively, at any time, or if the woman was under treatment with thyroid hormone supplementation due to overt hypothyroidism as defined above; if women were pre-treated, medical records providing TSH and T4 levels were obtained in order to verify the diagnosis of overt hypothyroidism), or (ii) subclinical hypothyroidism defined as TSH > 2.5 μIU/ml according to recent guidelines [15]. Notably, all patients who received thyroid hormone supplementation, regardless of whether this was due to overt or subclinical hypothyroidism, revealed a TSH level < 2.5 IU/mL at the time of insemination.

All serum parameters included in this analysis were obtained on the 3^rd^ to the 5^th^ day of the menstrual cycle with IUI. In our department, the normal range for TSH is 0.44–3.77 mU/ml, 0–34 IU/ml for anti-TPO antibodies, and 0–33 IU/ml for TG-Ab. These normal ranges had been calculated by the laboratory and all examined serum parameters were determined in the ISO-certified central laboratory of the General Hospital of Vienna, Vienna, Austria using commercially available assays (TSH: REF 11731459, measuring range 0.005-100 μIU/ml, Anti-TG: REF 06368697, measuring range 10.0-4000 IU/ml, Anti-TPO: REF 06368590, measuring range 5.0-600 IU/ml, cobas, Roche, Germany).

### Statistical analysis

Nominal variables are reported as numbers and frequencies, and continuous variables as medians and interquartile ranges (IQR). Statistical analysis was accomplished using (i) Wilcoxon-Mann-Whitney-tests for metric variables, (ii) chi-square- or Fisher’s exact tests for parametric variables, and (iii) a logistic regression model with Wald’s tests to test the statistical significance of all coefficients. Odds ratios (OR) are given, including the 95 per cent confidence interval (95% CI). P-values < 0.05 were considered statistically significant. Statistical analyses were performed with the SPSS software package, version 19 (SPSS, Chicago).

## Results

Detailed patient characteristics are provided in Table 1. There were no cases of hyperthyroidism. The clinical pregnancy rate was 6.9% (37/540). We focused on predictive factors for pregnancy (Table 2). In a univariate analysis, several parameters were found to be associated with a lower chance to achieve a pregnancy after IUI, namely, increasing age, TPO-Ab and TG-Ab levels above the upper level of normal, the need for thyroid medication due to overt hypothyroidism, and the presence of a male factor. In contrast, thyroid hormone supplementation for TSH levels exceeding the threshold of 2.5 μIU/ml, and CC stimulation and ovulation induction with HCG were associated with higher pregnancy rates. When these factors were tested in a multivariate analysis, only age (odds ratio 0.94, 95% CI 0.87-0.99), thyroid hormone supplementation for TSH levels exceeding the threshold of 2.5 μIU/ml (odds ratio 3.31, 95% CI 1.31-8.35), and ovulation induction with HCG (odds ratio 5.37, 95% CI 1.72-16.69) remained significantly predictive (p < 0.05; Table 2).

**Table 1:** Patient characteristics

**Characteristics**	
*Age (years)*^*a*^	34 (29-39)
*Body mass index (kg/m*^*2*^*)*^*a*^	22.9 (20.3-26.0)
*Primary infertility*^*b*^	277 (51.3)
*Parity*^*b*^	0 (0-0)
*TSH (μU/ml)*^*a*^	1.6 (1.2-2.3)
*TPO-Ab (IU/ml)*^*a*^	0.0 (0-6)
*TPO-Ab > upper level of normal*^*b*^	61 (11.3)
*TG-Ab (IU/ml)*^*a*^	0.0 (0-22)
*TG-Ab > upper level of normal*^*b*^	107 (19.8)
*Thyroid medication for overt hypothyroidism*^*b*^	97 (18.0)
*Thyroid medication TSH > 2.5 μIU/ml*^*b*^	71 (13.1)
*Unilateral tubal factor*^*b*^	42 (7.8)
*Presence of PCO-S*^*c*^	89 (16.5)
*Metformin treatment*^*b*^	34 (6.3)
*Clomifen citrate stimulation*^*b*^	165 (30.6)
*Ovulation induction with HCG*^*a*^	266 (49.3)
*Endometrial thickness*^*a*^	9.0 (8-10)
*Male factor*^*b*^	282 (52.2)

Data are provided as ^a^median (interquartile ranges) or ^b^number (frequencies).

**Table 2:** Univariate and multivariate analysis

	**Pregnancy (N = 37)**	**No pregnancy (N = 503)**	**Univariate analysis**		**Multivariate analysis**	
			**OR (95% CI)**^**a**^	**p**	**OR (95% CI)**^**a**^	**p**
Age (years)^b^	30 (30-35)	34 (29-39)	0.92 (0.87;0.98)	*0.015*^*d*^	0.94 (0.87;0.99)	*0.049*^*d*^
*Body mass index (kg/m*^*2*^*)*^*b*^	20.9 (19.9-27.0)	22.9 (20.4-26.0)	1.02 (0.93;1.07)	0.931	-	-
*Primary infertility (vs. secondary infertility)*^*c*^	19 (51.4)	258 (51.3)	1.00 (0.95;1.05)	0.861	-	-
*Parity*^*c*^	0 (0-1)	0 (0-0)	1.01 (0.60;1.71)	0.954	-	-
*TSH (μIU/ml)*^*b*^	1.6 (1.1-2.2)	1.9 (1.2-2.4)	0.89 (0.46;1.21)	0.556	-	-
*TPO-Ab > upper level of normal*^*c*^	0 (0)	61 (12.1)	0.07 (0.05;0.10)	*0.015*^*d*^	0 (0;inf)	0.997
*TG-Ab > upper level of normal*^*c*^	2 (5.4)	105 (20.9)	0.22 (0.05;0.91)	*0.037*^*d*^	0.87 (0.19;4.03)	0.861
*Thyroid medication for overt hypothyroidism*^*c*^	2 (5.4)	95 (18.9)	0.25 (0.06;1.04)	*0.036*^*d*^	0.54 (0.12;2.47)	0.338
*Thyroid medication TSH > 2.5 μIU/ml*^*c*^	16 (43.2)	55 (10.9)	6.94 (3.60;13.40)	*<0.001*^*d*^	3.31 (1.31;8.35)	*0.009*^*d*^
*Presence of PCO-S*^*c*^	8 (21.6)	81 (16.1)	0.71 (0.31;1.60)	0.407	-	-
*Metformin treatment*^*b*^	3 (8.1)	31 (6.2)	1.34 (0.31;4.92)	*0.500*	-	-
*Clomifen citrate stimulation*^*c*^	19 (51.4)	146 (29.0)	0.39 (0.20;0.77)	*0.006*^*d*^	0.77 (0.32;1.85)	0.629
*Number of IUI treatment cycle*^*b*^	1 (1-1)	1 (1-1)	0.90 (0.59;1.37)	0.638	-	-
*Ovulation induction with HCG*^*c*^	27 (73.0)	239 (47.5)	2.95 (1.40;6.22)	*0.005*^*d*^	5.37 (1.72;16.69)	*0.004*^*d*^
*Endometrial thickness*^*b*^	10 (8-11)	8 (9-10)	1.09 (0.91;1.31)	0.324	-	-
*Male factor*^*c*^	13 (35.1)	269 (53.5)	0.47 (0.23;0.95)	*0.034*^*d*^	0.60 (0.27;1.03)	0.067

^a^OR = odds ratio, 95% CI = 95% confidence interval.

^b^Continuous variable, provided in median (interquartile range).

^c^Nominal variable, provided in n (%).

^d^Italic numbers in p columns indicate statistical significance.

Based on these results, we chose to compare patients with thyroid medication for TSH levels exceeding the threshold of 2.5 μIU/ml (n = 71) and patients without any thyroid medication (n = 372). Patients who received thyroid hormone supplementation for initial TSH levels > 2.5 μIU/ml achieved pregnancy and revealed increased TPO-Ab and TG-Ab levels as well as polycystic ovary syndrome significantly more often (see Table 3). In a next step, patients with thyroid medication for TSH levels > 2.5 μIU/ml (n = 71) were compared to patients with thyroid medication for overt hypothyroidism (n = 97; Table 3). The latter were significantly older and revealed elevated TPO-Ab levels more often as well as a lower pregnancy rate.

**Table 3:** Comparison of patients with thyroid medication for TSH levels exceeding the threshold of 2.5 μIU/ml and patients without any thyroid medication

	**Thyroid medication for TSH levels**	**No thyroid medication**	**Thyroid medication for overt hypothyroidism**	**p**^**d**^	**p**^**e**^
	**> 2.5 μIU/ml (n = 71)**	**(n = 372)**	**(n = 97)**		
*Age (years)*^*a*^	33 (27-40)	33 (28-39)	34 (30-39)	0.324	*0.047*^*c*^
*Body mass index (kg/m*^*2*^*)*^*a*^	22.3 (19.4-25.1)	22.9 (20.3-26.7)	23.1 (21.4—25.1)	0.260	0.340
*Primary infertility (vs. secondary infertility)*^*b*^	36 (50.7)	202 (54.3)	39 (40.2)	0.578	0.176
*Parity*^*b*^	0 (0-1)	0 (0-1)	0 (0-1)	0.961	0.667
*TSH (μIU/ml)*^*a*^	1.6 (1.1-2.2)	1.5 (1.1-2.0)	1.4 (0.4-2.2)	0.422	0.107
*TPO-Ab > upper level of normal*^*b*^	8 (11.3)	14 (3.8)	39 (40.2)	*0.014*^*c*^	*< 0.001*^*c*^
*TG-Ab > upper level of normal*^*b*^	22 (31.0)	57 (15.3)	28 (28.9)	*0.004*^*c*^	0.767
*Presence of PCO-S*^*b*^	16 (22.5)	56 (15.1)	17 (17.5)	*< 0.001*^*c*^	0.419
*Metformin treatment*^*b*^	5 (7.0)	19 (5.1)	10 (10.3)	0.565	0.588
*Clomifen citrate stimulation*^*b*^	23 (32.4)	115 (30.9)	27 (27.8)	0.805	0.523
*Number of IUI treatment cycle*^*a*^	1 (1-1)	1 (1-1)	1 (1-1)	0.063	0.598
*Ovulation induction with HCG*^*b*^	36 (50.7)	181 (48.7)	49 (50.5)	0.752	0.981
*Endometrial thickness*^*a*^	9 (8-10)	9 (8-10)	9 (8-10)	0.866	0.864
*Male factor*^*b*^	35 (49.3)	195 (52.4)	52 (53.6)	0.629	0.581
*Pregnancy rate*^*b*^	16 (23.9)	19 (5.1)	2 (2.1)	*< 0.001*^*c*^	*< 0.001*^*c*^

Patients with overt hypothyroidism were excluded for this analysis.

^a^Continuous variable, provided in median (interquartile range).

^b^Nominal variable, provided in n (%).

^c^Italic letters indicate statistical significance.

^d^Comparison of patients with thyroid medication for TSH levels > 2.5 μIU/ml and patients without thyroid medication.

^e^Comparison of patients with thyroid medication for TSH levels > 2.5 μIU/ml and patients with thyroid medication for overt hypothyroidism.

Finally, we compared TSH levels before IUI in women with elevated (n = 61) and normal TPO-Ab levels (n = 479; 1.9 μIU/ml IQR 1.2-2.3 vs. 1.6 μIU/ml, IQR 1.1-2.0, respectively; p = 0.032) and in women with elevated (n = 107) and normal TG-Ab levels (n = 433; 1.5 μIU/ml, IQR 1.2-1.9 vs. 1.5 μIU/ml, IQR 1.2-2.0, respectively; p = 0.845).

## Discussion

The main findings of this retrospective study were the differences between the rates of IUI patients who received thyroid hormone supplementation for TSH levels exceeding the threshold of 2.5 μIU/ml in the pregnancy and the non-pregnancy groups. The rationale was to avoid any kind of subclinical hypothyroidism. The fact that these patients were also more likely to become pregnant after IUI when they were compared to patients without any thyroid hormone supplementation (Table 3) is one of the key findings of our study.

Notably, despite the lower pregnancy rate in patients with thyroid hormone supplementation for overt hypothyroidism than in those with supplementation for subclinical hypothyroidism, which can be explained by the lower age in the latter group (Table 3), a patient’s need for thyroid medication due to overt hypothyroidism was not a significant influence on IUI outcome in the multivariate model. This is in accordance with a recent study in IVF patients demonstrating that adequate levothyroxine treatment may overcome the detrimental effects of overt hypothyroidism [21]. These results were quite contradictory to a previous report suggesting the opposite [22]. When comparing the reports, it seems evident that in the study by Busnelli et al., thyrotropin serum levels below 2.5 μIU/ml were maintained, in contrast to a higher threshold of <4.0 μIU/ml in the study by Scoccia et al. These valuable results are supported by our data. Obviously, infertile women undergoing reproductive medicine procedures benefit from a more generous supplementation with levothyroxine, even in absence of overt hypothyroidism. A recent randomized study has demonstrated that levothyroxine treatment improved embryo quality and pregnancy outcome in subclinical hypothyroid women undergoing IVF [23].

One might argue that, in our study, in women who did not suffer from overt hypothyroidism, but who received thyroid supplementation for subclinical hypothyroidism with TSH levels > 2.5 μIU/ml, TSH levels might have been suppressed even more than in non-supplemented women. Hypothetically, this mechanism might have contributed to the increased pregnancy rates. However, we did not observe a difference in TSH levels on the 3^rd^ to the 5^th^ day of the menstrual cycle with IUI, neither in the multivariate model (Table 2) nor in the comparison of patients according to the type of thyroid hormone supplementation (for subclinical hypothyroidism/for overt hypothyroidism/patients without thyroid hormone supplementation; (Table 3). One could argue that thyroid hormone supplementation overcame fluctuations in thyroid hormone levels or other unknown effects that might have been due to HT [24]. This hypothesis is based on the following considerations: patients who were in need for thyroid hormone supplementation due to TSH levels > 2.5 μIU/ml revealed significantly more often increased thyroid autoantibody levels than patients who did not receive thyroid hormones at all (Table 3). This finding confirms the link between HT and subclinical hypothyroidism in our patient population. HT has been frequently discussed as a factor that negatively influences outcomes in infertility treatment [5,10,12,13]. However, higher anti-thyroid antibodies indicative of HT were associated with IUI failure after univariate analysis, but did not remain significant after multivariate analysis, in contrast to subclinical hypothyroidism treated with levothyroxine supplementation (Table 2). This suggests that (i) not HT – the most common cause of hypothyroidism in iodine-sufficient areas [3,4] – but rather, hypothyroidism itself is the more important thyroid factor that influences IUI outcome and (ii) thyroid hormone supplementation ameliorates hidden effects of HT on thyroid hormone levels. This is also supported by the fact that we found lower TSH levels in patients with than patients without elevated TPO-Ab despite the fact all women revealed TSH level < 2.5 μIU/ml at the time of IUI. However, it is unlikely that this explains the whole positive influence of thyroid hormone supplementation on pregnancy rates in our study. We find it hard to provide further explanations. It is evident that future studies are warranted to clarify the value of generous levothyroxine supplementation and the threshold that should be used to discriminate between infertile women in need and those not in need of such a treatment. Moreover, the difference between the upper limit of normal provided by the laboratory and the threshold considered to give levothyroxine supplementation in infertile women needs to be emphasized. This suggests that a specific range for women who plan to undergo assisted reproductive procedures could be needed in the future.

In addition to the above mentioned findings and considerations, the comparison of patients without any thyroid medication and patients with thyroid medication for TSH > 2.5 μIU/ml (Table 3) revealed that the latter achieved pregnancy more often and suffered from PCO-S more often. A previous report has already demonstrated a three-fold higher incidence of autoimmune thyroid diseases in women with PCO-S than in the general female population [25]. However, as demonstrated in Table 2, PCO-S did not independently contribute to the pregnancy rates after IUI.

Notably, ovulation induction with HCG was another factor that was significantly associated with clinical pregnancy after IUI. This is in accordance with previous studies [26,27]. After multivariate analysis, the parameter “male factor” differed only by trend (p = 0.067) between patients who achieved a clinical pregnancy and those who did not. This is likely due to the small number of patients in the pregnancy group. The presence of a male factor has frequently been reported as an influencing parameter on IUI outcome [28].

The study must be interpreted within its limitations: (i) its retrospective design; and (ii) the low pregnancy rate, which resulted in a small number of patients in the pregnancy group. In our study, the clinical pregnancy rate after IUI was 6.9%. This is comparatively low compared to the literature, which reports rates of about 10-20% [2,29]. Notably, in our department, many IUI procedures were performed according to the patient’s desire. This was the case for couples who could not afford an IVF treatment and opted for IUI, for example, in cases of infertility due to a moderate male factor, and for women aged 40 years or older. The latter is also highlighted by age distribution, as demonstrated in Table 2. Women who did not achieve pregnancy had a median age of 34 years, with an upper IQR level of 39. Moreover, increasing age remained a significant risk factor for IUI failure after multivariate analysis. This is in accordance with previous studies [29-32]. (iii) One of the most common reasons for hypothyroidism is iodine deficiency. Unfortunately, we cannot provide data on iodine status in our patients. However, since the second iodine prophylaxis, Austria is not to be considered a region of iodine deficiency any more. Moreover, all our patients were recommended to use common micronutrient supplementation preparations. These always include at least 100 μg iodine per day. Nonetheless, the lack of data on iodine status has to be considered a study limitation.

## Conclusions

Our study provides the following key findings: (i) “classic” predictive factors for IUI outcome, including age, ovulation induction with HCG and – although only differing by trend –the presence of a male factor were confirmed; (ii) women with overt hypothyroidism who received thyroid hormone supplementation that was sufficient to keep TSH levels < 2.5 μIU/ml were not at greater risk of IUI failure; and (iii) for women who received thyroid hormone supplementation due to subclinical hypothyroidism, with TSH levels > 2.5 μIU/ml, higher pregnancy rates were found after IUI. These findings suggest that women undergoing IUI, and, hypothetically, also other medical interventions for infertility, benefit from a strict thyroid hormone supplementation in order to achieve lower TSH levels. Although our study sheds new light on the issue of the influence of thyroid function and thyroid supplementation on IUI success, prospective studies are needed to confirm our results. Future work should also clarify whether the TSH threshold for thyroid hormone supplementation should be decreased for infertile women.

## Competing interests

All authors declare that there are no potential conflicts of interest, whether of a financial or other nature.

## Authors' contributions

All authors contributed to the writing process of the manuscript and approved the final version. BJ and JO were the principal investigators, wrote the study protocol and manuscript. AP, EV, KM and RP worked as co-investigators performed the literature search and were crucially involved in data interpretation. All authors read and approved the final manuscript.
